# Supplementary material for: Lack of Highly Pathogenic Avian Influenza H5N1 in the South Shetland Islands in Antarctica, Early 2023
Source: Animals (Basel). 2024 Mar 26;14(7):1008. doi: 10.3390/ani14071008 (PMC11011164; doi:10.3390/ani14071008)
Supplement: Supplementary file 1 [file animals-14-01008-s001.zip › animals-2807465-supplementary.pdf]

We gratefully acknowledge the authors, originating and submitting laboratories of the sequences from GISAID's EpiFlu™ Database on which this research is based. The list is detailed below. All submitters of data may be contacted directly via www.gisaid.org

Segment ID	Segment	Country	Collection date	Isolate-ID	Isolate name	Originating Lab	Submitting Lab	Authors
EPI2242291	HA	Germany	2022-Jun-30	EPI_ISL_16096040	A/arctic tern/Germany-SH/AI03798/2022	Landeslabor Schleswig-Holstein	Friedrich-Loeffler-Institut	
EPI2242295	NS	Germany	2022-Jun-30	EPI_ISL_16096040	A/arctic tern/Germany-SH/AI03798/2022	Landeslabor Schleswig-Holstein	Friedrich-Loeffler-Institut	
EPI2242296	PA	Germany	2022-Jun-30	EPI_ISL_16096040	A/arctic tern/Germany-SH/AI03798/2022	Landeslabor Schleswig-Holstein	Friedrich-Loeffler-Institut	
EPI2242297	PB1	Germany	2022-Jun-30	EPI_ISL_16096040	A/arctic tern/Germany-SH/AI03798/2022	Landeslabor Schleswig-Holstein	Friedrich-Loeffler-Institut	
EPI2242294	NP	Germany	2022-Jun-30	EPI_ISL_16096040	A/arctic tern/Germany-SH/AI03798/2022	Landeslabor Schleswig-Holstein	Friedrich-Loeffler-Institut	
EPI2242293	NA	Germany	2022-Jun-30	EPI_ISL_16096040	A/arctic tern/Germany-SH/AI03798/2022	Landeslabor Schleswig-Holstein	Friedrich-Loeffler-Institut	
EPI2242292	MP	Germany	2022-Jun-30	EPI_ISL_16096040	A/arctic tern/Germany-SH/AI03798/2022	Landeslabor Schleswig-Holstein	Friedrich-Loeffler-Institut	
EPI2242298	PB2	Germany	2022-Jun-30	EPI_ISL_16096040	A/arctic tern/Germany-SH/AI03798/2022	Landeslabor Schleswig-Holstein	Friedrich-Loeffler-Institut	
EPI2244092	NP	Denmark	2022-Jun-19	EPI_ISL_16112044	A/Arctic_tern/Denmark/05803-1.01/2022	Statens Serumm Institute	Statens Serum Institute	Hjulsager,Charlotte; Liang,Yuan
EPI2244088	PB2	Denmark	2022-Jun-19	EPI_ISL_16112044	A/Arctic_tern/Denmark/05803-1.01/2022	Statens Serumm Institute	Statens Serum Institute	Hjulsager,Charlotte; Liang,Yuan
EPI2244089	PB1	Denmark	2022-Jun-19	EPI_ISL_16112044	A/Arctic_tern/Denmark/05803-1.01/2022	Statens Serumm Institute	Statens Serum Institute	Hjulsager,Charlotte; Liang,Yuan
EPI2244090	PA	Denmark	2022-Jun-19	EPI_ISL_16112044	A/Arctic_tern/Denmark/05803-1.01/2022	Statens Serumm Institute	Statens Serum Institute	Hjulsager,Charlotte; Liang,Yuan
EPI2244091	HA	Denmark	2022-Jun-19	EPI_ISL_16112044	A/Arctic_tern/Denmark/05803-1.01/2022	Statens Serumm Institute	Statens Serum Institute	Hjulsager,Charlotte; Liang,Yuan
EPI2244093	NA	Denmark	2022-Jun-19	EPI_ISL_16112044	A/Arctic_tern/Denmark/05803-1.01/2022	Statens Serumm Institute	Statens Serum Institute	Hjulsager,Charlotte; Liang,Yuan
EPI2244094	MP	Denmark	2022-Jun-19	EPI_ISL_16112044	A/Arctic_tern/Denmark/05803-1.01/2022	Statens Serumm Institute	Statens Serum Institute	Hjulsager,Charlotte; Liang,Yuan
EPI2244095	NS	Denmark	2022-Jun-19	EPI_ISL_16112044	A/Arctic_tern/Denmark/05803-1.01/2022	Statens Serumm Institute	Statens Serum Institute	Hjulsager,Charlotte; Liang,Yuan
EPI2616105	NP	United Kingdo	2023-May-28	EPI_ISL_17971920	A/Arctic_Tern/Wales/076069/2023	Animal and Plant Health Agency (APHA)	Animal and Plant Health Agency (APHA)	
EPI2616106	NS	United Kingdo	2023-May-28	EPI_ISL_17971920	A/Arctic_Tern/Wales/076069/2023	Animal and Plant Health Agency (APHA)	Animal and Plant Health Agency (APHA)	
EPI2616107	MP	United Kingdo	2023-May-28	EPI_ISL_17971920	A/Arctic_Tern/Wales/076069/2023	Animal and Plant Health Agency (APHA)	Animal and Plant Health Agency (APHA)	
EPI2616108	PA	United Kingdo	2023-May-28	EPI_ISL_17971920	A/Arctic_Tern/Wales/076069/2023	Animal and Plant Health Agency (APHA)	Animal and Plant Health Agency (APHA)	
EPI2616109	PB2	United Kingdo	2023-May-28	EPI_ISL_17971920	A/Arctic_Tern/Wales/076069/2023	Animal and Plant Health Agency (APHA)	Animal and Plant Health Agency (APHA)	
EPI2616110	PB1	United Kingdo	2023-May-28	EPI_ISL_17971920	A/Arctic_Tern/Wales/076069/2023	Animal and Plant Health Agency (APHA)	Animal and Plant Health Agency (APHA)	
EPI2616111	NA	United Kingdo	2023-May-28	EPI_ISL_17971920	A/Arctic_Tern/Wales/076069/2023	Animal and Plant Health Agency (APHA)	Animal and Plant Health Agency (APHA)	
EPI2616112	HA	United Kingdo	2023-May-28	EPI_ISL_17971920	A/Arctic_Tern/Wales/076069/2023	Animal and Plant Health Agency (APHA)	Animal and Plant Health Agency (APHA)	
EPI2665335	PA	Netherlands	2023-Jul-13	EPI_ISL_18059060	A/Arctic Tern/Netherlands/1/2023	Erasmus Medical Center	Erasmus Medical Center	Vuong,O; Thewessen, S; Bellido-Martin, A.B.; Fouchier, R.A.M.
EPI2665332	NP	Netherlands	2023-Jul-13	EPI_ISL_18059060	A/Arctic Tern/Netherlands/1/2023	Erasmus Medical Center	Erasmus Medical Center	Vuong,O; Thewessen, S; Bellido-Martin, A.B.; Fouchier, R.A.M.
EPI2665333	NS	Netherlands	2023-Jul-13	EPI_ISL_18059060	A/Arctic Tern/Netherlands/1/2023	Erasmus Medical Center	Erasmus Medical Center	Vuong,O; Thewessen, S; Bellido-Martin, A.B.; Fouchier, R.A.M.
EPI2665334	MP	Netherlands	2023-Jul-13	EPI_ISL_18059060	A/Arctic Tern/Netherlands/1/2023	Erasmus Medical Center	Erasmus Medical Center	Vuong,O; Thewessen, S; Bellido-Martin, A.B.; Fouchier, R.A.M.
EPI2665336	PB2	Netherlands	2023-Jul-13	EPI_ISL_18059060	A/Arctic Tern/Netherlands/1/2023	Erasmus Medical Center	Erasmus Medical Center	Vuong,O; Thewessen, S; Bellido-Martin, A.B.; Fouchier, R.A.M.
EPI2665337	PB1	Netherlands	2023-Jul-13	EPI_ISL_18059060	A/Arctic Tern/Netherlands/1/2023	Erasmus Medical Center	Erasmus Medical Center	Vuong,O; Thewessen, S; Bellido-Martin, A.B.; Fouchier, R.A.M.
EPI2665338	NA	Netherlands	2023-Jul-13	EPI_ISL_18059060	A/Arctic Tern/Netherlands/1/2023	Erasmus Medical Center	Erasmus Medical Center	Vuong,O; Thewessen, S; Bellido-Martin, A.B.; Fouchier, R.A.M.
EPI2665339	HA	Netherlands	2023-Jul-13	EPI_ISL_18059060	A/Arctic Tern/Netherlands/1/2023	Erasmus Medical Center	Erasmus Medical Center	Vuong,O; Thewessen, S; Bellido-Martin, A.B.; Fouchier, R.A.M.
EPI2744792	NS	United Kingdo	2023-Jul-05	EPI_ISL_18258046	A/arctic_tern/Scotland/092455/2023	Animal and Plant Health Agency (APHA)	Animal and Plant Health Agency (APHA)	
EPI2744793	MP	United Kingdo	2023-Jul-05	EPI_ISL_18258046	A/arctic_tern/Scotland/092455/2023	Animal and Plant Health Agency (APHA)	Animal and Plant Health Agency (APHA)	
EPI2744794	PA	United Kingdo	2023-Jul-05	EPI_ISL_18258046	A/arctic_tern/Scotland/092455/2023	Animal and Plant Health Agency (APHA)	Animal and Plant Health Agency (APHA)	
EPI2744795	PB2	United Kingdo	2023-Jul-05	EPI_ISL_18258046	A/arctic_tern/Scotland/092455/2023	Animal and Plant Health Agency (APHA)	Animal and Plant Health Agency (APHA)	
EPI2744796	PB1	United Kingdo	2023-Jul-05	EPI_ISL_18258046	A/arctic_tern/Scotland/092455/2023	Animal and Plant Health Agency (APHA)	Animal and Plant Health Agency (APHA)	
EPI2744791	NP	United Kingdo	2023-Jul-05	EPI_ISL_18258046	A/arctic_tern/Scotland/092455/2023	Animal and Plant Health Agency (APHA)	Animal and Plant Health Agency (APHA)	
EPI2744797	NA	United Kingdo	2023-Jul-05	EPI_ISL_18258046	A/arctic_tern/Scotland/092455/2023	Animal and Plant Health Agency (APHA)	Animal and Plant Health Agency (APHA)	
EPI2744798	HA	United Kingdo	2023-Jul-05	EPI_ISL_18258046	A/arctic_tern/Scotland/092455/2023	Animal and Plant Health Agency (APHA)	Animal and Plant Health Agency (APHA)	
EPI2760797	NP	United Kingdo	2023-Jul-13	EPI_ISL_18325134	A/Arctic_Tern/England/402279/2023	Animal and Plant Health Agency (APHA)	Animal and Plant Health Agency (APHA)	
EPI2760798	NS	United Kingdo	2023-Jul-13	EPI_ISL_18325134	A/Arctic_Tern/England/402279/2023	Animal and Plant Health Agency (APHA)	Animal and Plant Health Agency (APHA)	
EPI2760799	MP	United Kingdo	2023-Jul-13	EPI_ISL_18325134	A/Arctic_Tern/England/402279/2023	Animal and Plant Health Agency (APHA)	Animal and Plant Health Agency (APHA)	
EPI2760800	PA	United Kingdo	2023-Jul-13	EPI_ISL_18325134	A/Arctic_Tern/England/402279/2023	Animal and Plant Health Agency (APHA)	Animal and Plant Health Agency (APHA)	
EPI2760801	PB2	United Kingdo	2023-Jul-13	EPI_ISL_18325134	A/Arctic_Tern/England/402279/2023	Animal and Plant Health Agency (APHA)	Animal and Plant Health Agency (APHA)	
EPI2760802	PB1	United Kingdo	2023-Jul-13	EPI_ISL_18325134	A/Arctic_Tern/England/402279/2023	Animal and Plant Health Agency (APHA)	Animal and Plant Health Agency (APHA)	
EPI2760803	NA	United Kingdo	2023-Jul-13	EPI_ISL_18325134	A/Arctic_Tern/England/402279/2023	Animal and Plant Health Agency (APHA)	Animal and Plant Health Agency (APHA)	
EPI2760804	HA	United Kingdo	2023-Jul-13	EPI_ISL_18325134	A/Arctic_Tern/England/402279/2023	Animal and Plant Health Agency (APHA)	Animal and Plant Health Agency (APHA)	
